# The Effect of TGF-β on Treg Cells in Adverse Pregnancy Outcome upon *Toxoplasma gondii* Infection

**DOI:** 10.3389/fmicb.2017.00901

**Published:** 2017-05-26

**Authors:** Mingdong Zhao, Haixia Zhang, Xianbing Liu, Yuzhu Jiang, Liqin Ren, Xuemei Hu

**Affiliations:** ^1^Department of Radiology, Affiliated Hospital of Binzhou Medical UniversityBinzhou, China; ^2^Department of Immunology, Binzhou Medical UniversityYantai, China; ^3^Medicine and Pharmacy Research Center, Binzhou Medical UniversityYantai, China

**Keywords:** TGF-β, pSmad3, CTLA-4, PD-1, Treg cells, *Toxoplasma gondii*, pregnancy outcome

## Abstract

*Toxoplasma gondii* (*T. gondii*) is a ubiquitous intracellular protozoan parasite that causes adverse pregnancy outcomes. Its infection downregulates the Treg cell population and TGF-β level, which may contribute to adverse pregnancy outcomes. TGF-β plays a critical role in Treg cell development, but whether TGF-β treatment can affect the number and function of Treg cells and hence alleviate adverse pregnancy outcomes caused by *T. gondii* infection remains elusive. In this study, *T. gondii*-infected pregnant mice were treated with TGF-β or TGF-β-neutralizing antibody. The pregnancy outcomes were observed on gestational day 14. The numbers of Treg cells and pSmad3, programmed death 1 (PD-1), and Cytotoxic T lymphocyte-associated antigen-4 (CTLA-4) expression of Treg cells were analyzed by flow cytometry. Histological changes were assessed using HE staining, while IL-10 and TNF-α levels were measured using ELISA. The results indicated that TGF-β treatment improved the *T. gondii*-induced adverse pregnancy outcomes, with alleviation of hemorrhage, restoration of uterine spiral arteries of the placenta, and increased Treg cell numbers; meanwhile, TGF-β neutralization resulted in more serious adverse pregnancy outcomes, with serious hemorrhage, more dilated uterine spiral arteries, and decreased Treg cell numbers. pSmad3 expression in CD4^+^ cells and CTLA-4 and PD-1 levels on Treg cells were upregulated by TGF-β treatment, but downregulated by TGF-β neutralization. The ratio of IL-10/TNF-α also increased after TGF-β treatment, but decreased after TGF-β neutralization. Our data indicate that TGF-β treatment could improve adverse pregnancy outcomes caused by *T. gondii* infection by upregulating Treg cell differentiation and function via the TGF-β/Smad3 signaling pathway, but not the proliferation of Treg cells.

## Introduction

*Toxoplasma gondii (T. gondii)* is a widespread obligate intracellular protozoan parasite that can infect all warm-blooded animals (Leng et al., 2009). During gestation, *T. gondii* infection may cause maternal immune deregulation, particularly in early pregnancy, which can lead to adverse pregnancy outcomes, such as miscarriage, stillbirth, or fetal teratogenesis (Wang et al., 2011). Unfortunately, there is still no effective treatment for congenital toxoplasmosis. Therefore, strategies should be explored to alleviate the abnormal pregnancy outcomes caused by *T. gondii* infection.

Regulatory T (Treg) cells are vital to maternal–fetal tolerance. Evidence indicates that the number of Treg cells increases during normal pregnancy, while the impairment of Treg cells leads to adverse pregnancy outcomes (Aluvihare et al., 2004). Our previous study demonstrated that the population of Treg cells was downregulated in the placentas and spleens of *T. gondii*-infected mice (Zhang H. et al., 2012). Transferring placental Treg cells enriched from normal pregnant mice into *T. gondii*-infected mice could improve the adverse pregnancy outcomes (Liu Y. et al., 2014). Therefore, maintaining a certain number of Treg cells is important for ameliorating the adverse effects of *T. gondii* infection on pregnancy.

TGF-β plays a critical role in the development of Treg cells, as CD4^+^ T cells deficient in TGF-β signaling cannot be converted into Treg cells *in vitro* or *in vivo* (Liu et al., 2008). Smad3 is essential for the TGF-β-mediated induction of Treg cells, which was revealed by analyzing Foxp3 expression in Smad3-deficient mice (Jana et al., 2009; Takimoto et al., 2010). Therefore, Treg cell development is regulated through the TGF-β/Smad3 pathway (Massagué and Wotton, 2000). Further evidence indicated that human umbilical cord blood-derived stromal cells, which secrete a high level of TGF-β, can modulate Foxp3 expression in Treg cells through the TGF-β/Smad3 pathway and regulate graft-versus-host disease (Zhang C. et al., 2012). TGF-β-induced Treg cells also play an important role in maintaining normal pregnancy, as the adoptive transfer of TGF-β-induced Treg cells can prevent spontaneous abortion in mice (Qiu et al., 2015). However, our previous studies demonstrated that *T. gondii* infection decreased TGF-β levels at the maternal–fetal interface of pregnant mice (Zhang H. et al., 2012; Liu Y. et al., 2014). Nonetheless, whether the administration of TGF-β could affect Treg cell differentiation by the TGF-β/Smad3 pathway in *T. gondii*-infected mice and alleviate the adverse pregnancy outcomes remains unclear.

Cytotoxic T lymphocyte-associated antigen-4 (CTLA-4) and programmed death 1 (PD-1) are two key molecules for Treg cell functions (Freeman et al., 2000; Wing et al., 2008). Our previous study showed that the absolute numbers of CTLA-4^+^Tregs and PD-1^+^Tregs were decreased in *T. gondii*-infected pregnant mice (Liu Y. et al., 2014). Research showed that TGF-β can upregulate CTLA-4 expression during the induction of human peripheral naïve T cells to Treg cells (Yamagiwa et al., 2001). Whether TGF-β treatment could upregulate CTLA-4 and/or PD-1 expression on Treg cells in *T. gondii*-infected pregnant mice requires further exploration.

Therefore, this study explored whether TGF-β treatment could influence the differentiation and function of Treg cells via the TGF-β/Smad3 signaling pathway and consequently alleviate the abnormal pregnancy outcomes caused by *T. gondii* infection.

## Materials and Methods

### Animals

Prompted by reports that indicated that C57BL/6 mice are more susceptible than BALB/c mice to *T. gondii* infection (Schlüter et al., 1999), we used C57BL/6 females mated with BALB/c males as the pregnancy model. C57BL/6 female and BALB/c male mice aged 6–12 weeks old were allowed to reproduce and maintained in a specific pathogen-free environment. Female mice were mated with males overnight and checked for vaginal plugs in the morning. The mice with a vaginal plug [gestational day (gd) 0] were randomized into four groups, guaranteeing six pregnant mice eligible for each group: normal pregnant (N.P.) mice, infected pregnant (I.P.) mice, infected pregnant mice with TGF-β treatment (I.P.+TGF-β), and infected pregnant mice with TGF-β neutralization (I.P.+TGF-β neutralization). This study was carried out in accordance with the recommendations of the institutional animal experimental ethics committee of Binzhou Medical University. The protocol was approved by the biosafety committee of Binzhou Medical University.

### Infection and Treatment

*Toxoplasma gondii* tachyzoites were maintained in HEp-2 cells in MEM. Tachyzoites for the experiments were prepared by centrifugation of the supernatant of cell culture and resuspension of the parasites in phosphate-buffered solution (PBS). Pregnant mice were inoculated via I.P. injection with 400 tachyzoites in 200 μl of aseptic PBS on gd 8. The normal control group was inoculated with 200 μl of aseptic PBS at the same time. The infected mice received recombinant human TGF-β (0.75 μg in 200 μl of PBS; clone: 2Ar2; Abcam, United Kingdom) or TGF-β-neutralizing antibody (5 μg in 200 μl of PBS; clone: ab64715; Abcam, United Kingdom), all without LPS or any carrier, or the same volume of sterile PBS via tail vein injection on gd 7 and gd 9. The volume of TGF-β or TGF-β-neutralizing antibody injected in the mice was determined with reference to a previous study (Zhang et al., 2010), as well as our preparatory experiments.

### Pregnancy Outcomes

The mice were sacrificed on gd 14, their uteri were stripped, and the resorption rates were calculated as the ratio of abortion sites to the total number of nidation sites. The abortion sites were defined macroscopically by small and dissolved embryo, and the placentas were characterized for necroses and a hemorrhagic appearance. The fetal weights were recorded. The analyses of resorption rates and fetal weights of the four groups were conducted in a blinded manner.

### Flow Cytometry

Cell suspensions were prepared from the spleen or a mixture of all of the placentas and uteri, except one placenta for cytokine assays and one for histopathology assays. Mononuclear cells of the spleen or the mixture of placentas and uteri were obtained using mouse lymphocyte separation liquid (TBD Tianjin hao yang, China) with the density of 1.0830 ± 0.0001 g/ml (20°C) and washed in cold PBS. Cells were incubated with anti-CD4-Percp-cy5.5, anti-PD-1-PE, or anti-CTLA-4-PE at 4°C in the dark for 30 min, and then were washed twice. All of the above mAbs were from BD Pharmingen (United States). For intracellular Foxp3, pSmad3, and Ki-67 staining, cells were fixed and permeabilized in Fix/Perm buffer (eBioscience, United States) for 30 min and subsequently incubated with anti-Foxp3-APC, anti-pSmad3-PE, or anti-Ki-67-PE, in accordance with the manufacturer’s instructions. These mAbs were from eBioscience (United States). After washing three times with PBS, the cells were analyzed with a BD FACSCantoTM II flow cytometer (Becton Dickinson, United States) using BD FACSDiva 7.0 software.

### Histopathology

Hematoxylin and eosin (HE) staining was performed on sections from 4% paraformaldehyde-fixed placentas. Briefly, paraffin-embedded sections were cut to a thickness of 4.5 μm and then stained with HE. Each section was examined at 200× magnification with an Olympus microscope. Areas of hemorrhages and the diameter of uterine arteries in a 200× field were measured a blinded manner.

### Cytokine Assays

A placenta from each pregnant mouse was put into a test tube, to which homogenate buffer of 2 times the placenta weight was added; they were then comprehensively disrupted with a grinder and centrifuged at 12,000 *g* and 4°C for 30 min. The supernatants were collected and stored at -20°C. IL-10 (assay range: 30–700 pg/ml) and TNF-α (assay range: 25–500 pg/ml) were determined using ELISA kits. A total of 40 μl of dilution buffer was added to 10 μl of supernatant per test with each sample in triplicate, in accordance with the manufacturer’s protocols. All ELISA kits were from R&D Systems.

### Data Analysis and Statistics

Statistical analyses were performed using a statistics software package (SPSS 17.0). Data are presented as mean ± SD. Unpaired *t*-tests were used to compare two independent groups. One-way ANOVA was used to compare three independent groups. Differences at *p* < 0.05 or <0.01 were considered significant or very significant, respectively.

## Results

### TGF-β Improves Adverse Pregnancy Outcomes in Mice Infected with *T. gondii*

**FIGURE 1:**
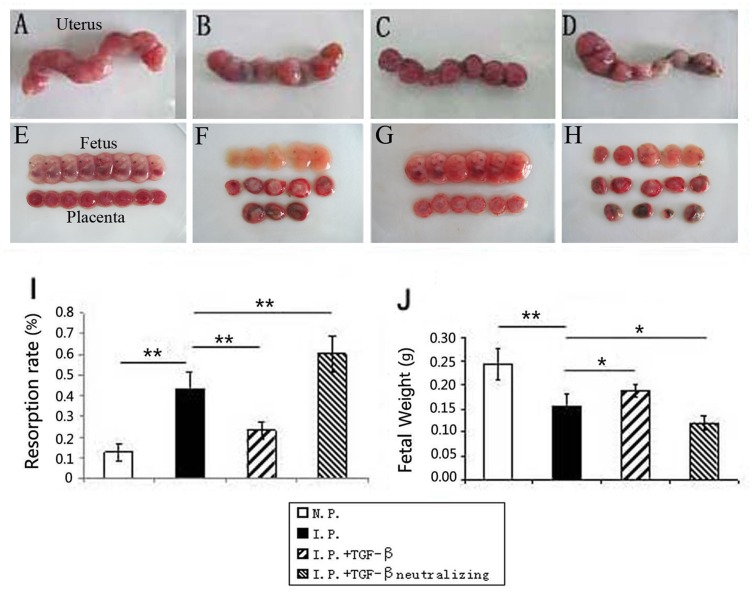
**The effect of TGF-β on the adverse pregnancy outcomes caused by *T. gondii* infection**. Representative pictures of the uterus, fetus, and placenta from normal pregnant mice **(A,E)**, infected mice **(B,F)**, TGF-β-treated mice **(C,G)**, and TGF-β-neutralized mice **(D,H)** at gd 14. **(I)** The resorption rate decreased after TGF-β treatment but increased after TGF-β neutralization compared with that in infected controls. **(J)** Fetal weight increased after TGF-β treatment but decreased after TGF-β neutralization. N.P.: normal pregnant mouse; I.P.: infected pregnant mouse; I.P. + TGF-β: infected pregnant mouse treated with TGF-β; I.P. + TGF-β neutralization: infected pregnant mouse treated with TGF-β-neutralizing antibody. Data are presented as the mean ± SEM (^∗^*p* < 0.05, ^∗∗^*p* < 0.01). Each group contained six mice.

As previously reported, *T. gondii* infection caused abnormal pregnancy outcomes (**Figures 1B,F**) compared with those in normal pregnant mice (**Figures 1A,E**). The adverse pregnancy outcomes were alleviated after TGF-β treatment (**Figures 1C,G**), evidenced by a lower resorption rate (**Figure 1I**) and a higher fetal weight (**Figure 1J**). To investigate further the potential relationship between TGF-β treatment and the improvement in adverse pregnancy outcomes, we performed TGF-β neutralization by treating infected mice with neutralizing antibody. Worse pregnancy outcomes were observed after TGF-β neutralization (**Figures 1D,H**), evidenced by a dramatically increased resorption rate (**Figure 1I**) and a lower fetal weight (**Figure 1J**).

### TGF-β Reduces the Inflammation Caused by *T. gondii* Infection at the Maternal–Fetal Interface

**FIGURE 2:**
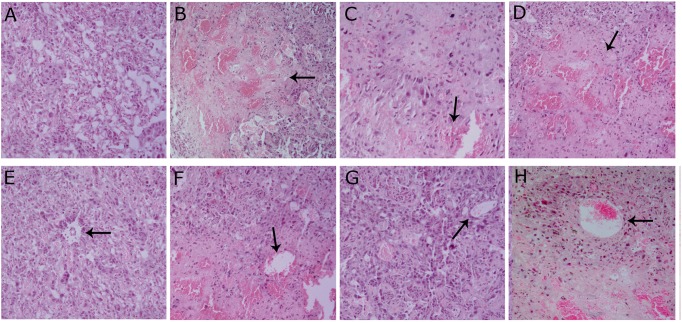
**Histopathological analysis of the placenta**. Uninfected pregnant mice showed normal placentas on gd 14 **(A)** and the uterine spiral arteries were not dilated **(E)**. Infected mice had more hemorrhage **(B)** and the uterine spiral arteries were dilated **(F)**. Infected mice treated with TGF-β had less hemorrhage **(C)** and the uterine spiral arteries were not patently dilated **(G)**. Infected mice treated with TGF-β-neutralizing antibody had more hemorrhage **(D)** and the uterine spiral arteries were obviously dilated **(H)**. Arrows showed hemorrhage **(B–D)** and the uterine spiral arteries **(E–H)**. The tissue sections were collected at gd 14, processed, and stained with H & E. The pictures are from 200× microscopic fields. Each group contained six mice.

Pathological histology analysis showed serious hemorrhage (**Figure 2B**) and dilated uterine spiral arteries (**Figure 2F**) in the placentas of infected mice, in contrast to those of uninfected mice (**Figures 2A,E**). TGF-β treatment alleviated the hemorrhage (**Figure 2C**) and partly restored the uterine spiral arteries (**Figure 2G**) compared with those in infected mice. In contrast, TGF-β neutralization resulted in serious hemorrhage (**Figure 2D**) and more dilated uterine spiral arteries of the placenta (**Figure 2H**). The improvement of inflammation by TGF-β was confirmed by measuring the changes of areas of hemorrhage and diameters of uterine arteries in the four groups, as listed in **Table 1**.

**Table 1:** Effect of TGF-β on inflammation at the maternal–fetal interface.

Groups	Mice	Inflammation areas (×10^3^μm^2^)	Diameters of uterine spiral arteries (μm)
N.P.	6	0	38.7 ± 4.4
I.P.	6	209.4 ± 38.4	66.0 ± 8.7^∗∗^
I.P.+ TGF-β	6	37.2 ± 13.2^∗∗∗^	49.3 ± 3.8^∗^
I.P.+ TGF-β–neutralization	6	327.4 ± 28.9^∗∗^	91.0 ± 10.8^∗^

*^∗^*p* < 0.05, ^∗∗^*p* < 0.01, ^∗∗∗^*p* < 0.001 by Unpaired *t*-test*.

### TGF-β Increases the Number of Treg Cells in Mice Infected with *T. gondii*

**FIGURE 3:**
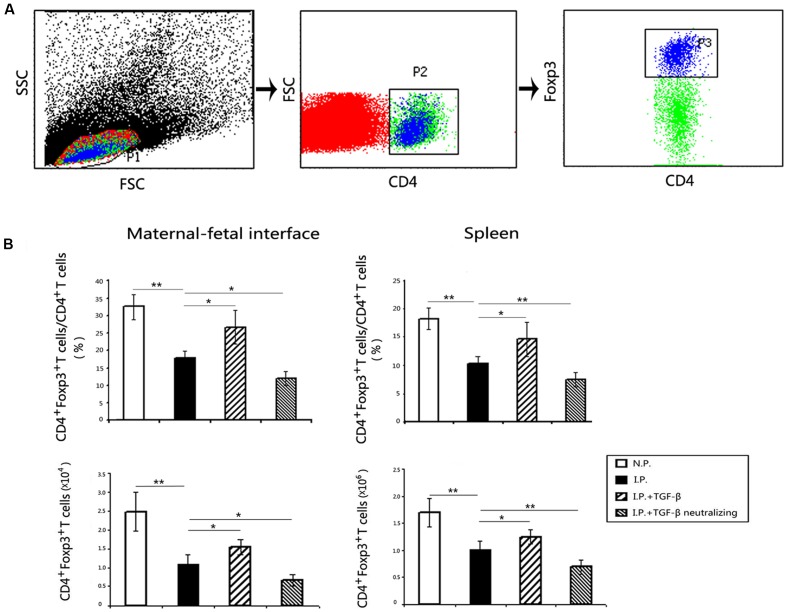
**Changes of the numbers of Treg cells at the maternal–fetal interface and in the spleen**. The process of Treg cell gating were presented **(A)**, and P2 was the CD4^+^T cells, P3 was CD4^+^Foxp3^+^ Treg cells. The percentage of Treg cells in CD4^+^T cells and the absolute numbers of Treg cells all decreased significantly after *T. gondii* infection at the maternal–fetal interface and in the spleen **(B)**. Importantly, Treg cell numbers increased in the TGF-β treatment group but decreased in the TGF-β neutralization group compared with those in infected controls. Data are presented as the mean ± SEM (^∗^*p* < 0.05, ^∗∗^*p* < 0.01). Each group contained six mice.

Our previous studies showed that both the level of placental TGF-β and the number of Treg cells decreased in *T. gondii*-infected mice. It is well known that TGF-β is a key cytokine in Treg cell development. To explore whether TGF-β treatment would boost the population of Treg cells, the numbers of Treg cells in the spleen or in the mixture of placentas and uteri of mice were assessed using flow cytometry. The results showed that TGF-β treatment upregulated the Treg population in the placenta and uterus and in the spleen of infected mice (**Figure 3**), while TGF-β neutralization decreased the numbers of Treg cells in infected mice compared with those in controls.

### TGF-β Upregulates pSmad3 Expression in Mice Infected with *T. gondii*

**FIGURE 4:**
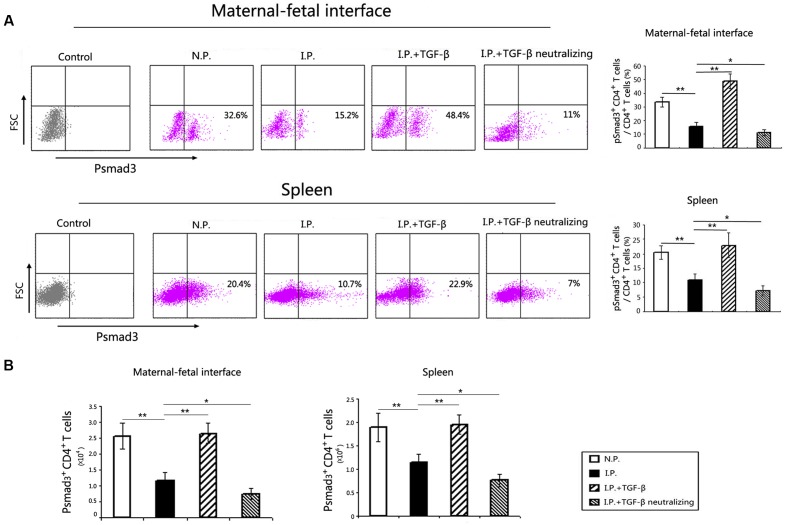
**pSmad3 expression at the maternal–fetal interface and in the spleen**. pSmad3 detection was gated on P2, the CD4^+^ T cells, as showed in **Figure 3**. Representative flow cytometry histograms showed the percentages of pSmad3^+^CD4^+^cells in CD4^+^ cells of the maternal–fetal interface and spleen **(A)**. Compared with that in normal controls, the proportion of pSmad3^+^CD4^+^cells among CD4^+^ cells was decreased at the maternal–fetal interface and in the spleen of the infected mice. Compared with that in infected controls, the proportion of pSmad3^+^CD4^+^cells increased after TGF-β treatment, but decreased after treatment with TGF-β-neutralizing antibody, at the maternal–fetal interface and in the spleen. The absolute numbers of pSmad3^+^CD4^+^cells in the maternal–fetal interface and spleen were calculated **(B)**. The changes of the absolute numbers of pSmad3^+^CD4^+^cells were similar to that of the proportions among the four groups. Data are presented as the mean ± SEM (^∗^*p* < 0.05, ^∗∗^*p* < 0.01). Each group contained six mice.

It has been reported that the differentiation of Treg cells from naïve T cells is regulated primarily by the TGF-β/Smad pathway. To test the further mechanism of Treg cell expansion caused by TGF-β treatment of *T. gondii*-infected mice, we detected pSmad3 expression in CD4^+^ cells in the placenta and uterus and in the spleen. The results showed that the proportions of pSmad3^+^CD4^+^cells among CD4^+^ cells (**Figure 4A**), as well as the absolute numbers of pSmad3^+^CD4^+^cells (**Figure 4B**), decreased in the placenta and uterus and in the spleen in the mice infected with *T. gondii*. More importantly, TGF-β treatment upregulated pSmad3 expression, while TGF-β neutralization exacerbated the loss of pSmad3 locally and systemically.

### TGF-β Did Not Enhance the Proliferative Capacity of Treg Cells in the Mice Infected with *T. gondii*

**FIGURE 5:**
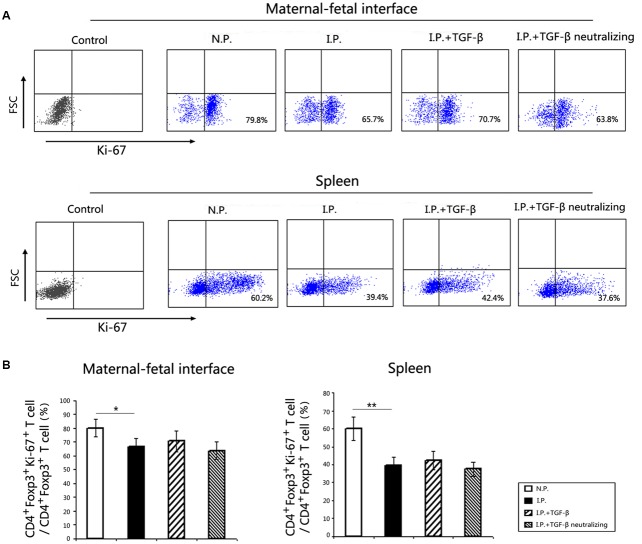
**The proliferative capacity of Treg cells at the maternal–fetal interface and in the spleen**. Ki-67 detection was gated on P3, the CD4^+^Foxp3^+^ Treg cells, as showed in **Figure 3**. The proportions of CD4^+^Foxp3^+^Ki-67^+^ cells among CD4^+^Foxp3^+^ Treg cells at the maternal–fetal interface **(A)** and in the spleen **(B)** are shown. The proportions of Ki-67^+^Treg cells were significantly reduced in *T. gondii*-infected mice compared with those in normal controls. However, there were no obvious changes in Ki-67 expression of Treg cells after TGF-β treatment or TGF-β neutralization compared to that in infected mice. Data are presented as the mean ± SEM (^∗^*p* < 0.05, ^∗∗^*p* < 0.01). Each group contained six mice.

To determine whether Treg cell proliferation contributes to the enlarged population of Treg cells, we evaluated the proliferative capacity of Treg cells by staining Ki-67 of Treg cells. The proportions of CD4^+^Foxp3^+^Ki-67^+^ cells among CD4^+^Foxp3^+^ Treg cells were significantly reduced in the placenta and uterus (**Figure 5A**) and in the spleen (**Figure 5B**) in infected mice compared with those in uninfected controls. However, TGF-β treatment, as well as TGF-β neutralization, failed to affect the Ki-67 expression of Treg cells. This meant that the boosted Treg cell population was attributable to the differentiation but not the proliferation of Treg cells.

### TGF-β Upregulates CTLA-4 and PD-1 Expression on Treg Cells in Mice Infected with *T. gondii*

**FIGURE 6:**
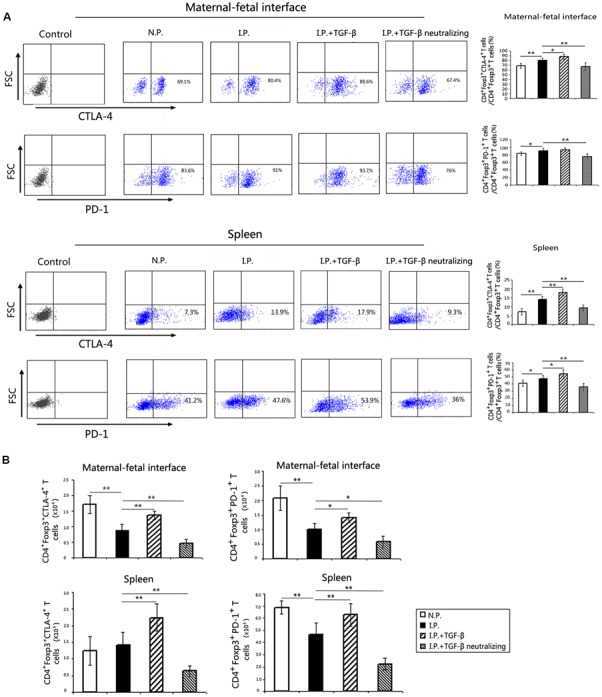
**PD-1 and CTLA-4 expression on Treg cells**. The detection of PD1 and CTLA-4 were gated on P3, the CD4^+^Foxp3^+^ Treg cells, as showed in **Figure 3**. **(A)** Representative flow cytometry histograms of PD-1 and CTLA-4 from the maternal–fetal interface and the spleen are presented. At the maternal–fetal interface and in the spleen, the proportions of PD-1^+^ Treg cells among Treg cells were increased in the infected group compared with those in the normal group. Compared with those in the infected group, the proportions of CTLA-4^+^Treg cells and PD-1^+^Treg cells in the spleen and the proportions of CTLA-4^+^Treg cells in the placenta and uterus were all upregulated after TGF-β treatment and reduced after TGF-β neutralization compared with those in the infected group. **(B)** The absolute numbers of CTLA-4^+^Treg cells and PD-1^+^Treg cells were all increased in the placenta and uterus and in the spleen from mice with TGF-β treatment, while they decreased in the mice with TGF-β neutralization. Data are presented as the mean ± SEM (^∗^*p* < 0.05, ^∗∗^*p* < 0.01). Each group contained six mice.

A study has shown that TGF-β influences the suppressive function of Treg cells (Marie et al., 2005). Therefore, we wanted to explore whether TGF-β treatment could affect the expression of CTLA-4 and PD-1 on Treg cells. The results indicated that the percentages of CD4^+^Foxp3^+^PD1^+^ cells to CD4^+^Foxp3^+^ cells and the percentages of CD4^+^Foxp3^+^CTLA-4^+^ cells to CD4^+^Foxp3^+^ cells of the placenta and uterus was upregulated in the group with TGF-β treatment, and downregulated in the group with TGF-β neutralization, compared with the levels in the infected group (**Figure 6A**). Furthermore, we found that the absolute numbers of CTLA-4^+^Treg cells and PD-1^+^Treg cells were all increased in the placenta and uterus and in the spleen of mice with TGF-β treatment, while they decreased in the mice with TGF-β neutralization (**Figure 6B**).

### The Imbalance between TNF-α and IL-10 in Mice Caused by *T. gondii* Infection Was Rectified by TGF-β

**FIGURE 7:**
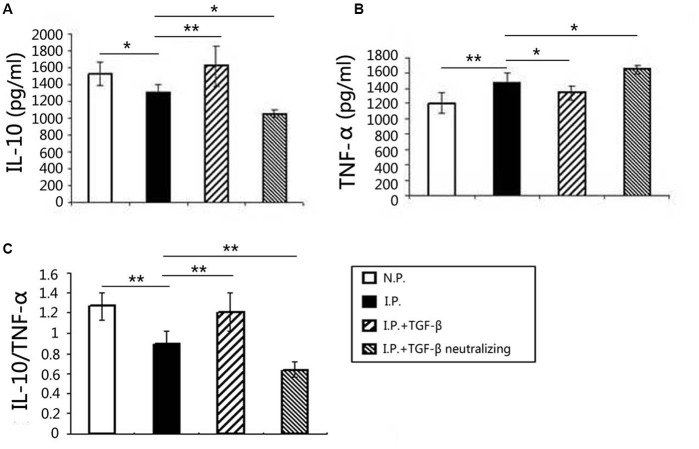
**Changes in the cytokine milieu at the maternal–fetal interface**. The levels of IL-10 and TNF-α in the placenta and uterus were measured using ELISA. Compared with those in the normal group, the IL-10 level **(A)** decreased, the TNF-α level **(B)** increased, and the ratio of IL-10/TNF-α **(C)** decreased after *T. gondii* infection. Compared with those in the infected group, the IL-10 level increased, the TNF-α level decreased, and the ratio of IL-10/TNF-α increased in the TGF-β treatment group. In contrast, the IL-10 level decreased, the TNF-α level increased, and the IL-10/TNF-α ratio decreased in the TGF-β neutralization group. Data are presented as the mean ± SEM (^∗^*p* < 0.05, ^∗∗^*p* < 0.01). Each group contained six mice.

To evaluate the changes in the cytokine milieu after TGF-β or TGF-β-neutralizing antibody treatment, the IL-10 and TNF-α expression levels at the maternal–fetal interface were measured by ELISA. Compared with those in normal controls, IL-10 expression decreased (**Figure 7A**) and TNF-α expression increased (**Figure 7B**) after *T. gondii* infection, so the ratio of IL-10/TNF-α (**Figure 7C**) decreased. Compared with those in infected controls, the IL-10 level was elevated but the TNF-α level was diminished; correspondingly, the ratio of IL-10/TNF-α increased after TGF-β treatment. In contrast, after TGF-β neutralization, IL-10 levels decreased and TNF-α levels increased, so the IL-10/TNF-α ratio decreased.

## Discussion

Our previous studies have shown that the abnormal pregnancy outcomes caused by *T. gondii* infection are associated with an abnormal immuno-microenvironment, which contains immunocytes and cytokines, at the maternal–fetal interface (Zhang H. et al., 2012; Xu et al., 2013; Liu X. et al., 2014; Liu Y. et al., 2014). We previously reported that the placental TGF-β level was decreased in mice with adverse pregnancy outcomes after *T. gondii* infection (Zhang H. et al., 2012; Liu Y. et al., 2014). However, whether TGF-β treatment could alleviate the adverse pregnancy outcomes associated with *T. gondii* infection remains unclear. In this study, we treated *T. gondii*-infected mice with recombinant TGF-β. The results showed that TGF-β treatment improved the pregnancy outcomes of *T. gondii*-infected mice and that this improvement was accompanied by decreased resorption rates and increased fetal weights. To investigate the potential link between TGF-β treatment and the improvement of adverse pregnancy outcomes further, we evaluated pregnancy outcomes in infected mice treated with TGF-β-neutralizing antibodies. The infected mice treated with TGF-β-neutralizing antibodies showed more serious pregnancy outcomes, with increased resorption rates and reduced fetal weights. This indicated that TGF-β treatment indeed improved the abnormal pregnancy outcomes induced by *T. gondii* infection.

To evaluate further the mechanism by which TGF-β treatment improved the adverse pregnancy outcomes associated with *T. gondii* infection, we analyzed changes in the inflammatory response of the placenta. The pathological histology analysis showed less hemorrhage or dilated uterine spiral arteries in the placentas after TGF-β treatment, while hemorrhage and dilated uterine spiral arteries were more obvious after TGF-β neutralization. Our results indicate that TGF-β treatment can suppress the high levels of inflammatory response at the maternal–fetal interface caused by *T. gondii* infection and benefit the development of the fetus.

It has been reported that a certain number of Treg cells are imperative for normal pregnancy (Sasaki et al., 2004; Prins et al., 2009). Studies have shown that TGF-β could induce the conversion of CD4^+^ T cells to CD4^+^Foxp3^+^ Treg cells through the TGF-β/Smad3 pathway (Massagué and Wotton, 2000; Jana et al., 2009; Zhang C. et al., 2012). In the present study, we found that TGF-β treatment indeed increased the absolute number of Treg cells in the placenta and uterus and in the spleen, while TGF-β neutralization decreased the number of Treg cells. Therefore, TGF-β treatment could ameliorate the adverse pregnancy outcomes by increasing the number of Treg cells locally and systemically. Accordingly, we determined the level of pSmad3 expression. The pSmad3 expression in CD4^+^ T cells was diminished in *T. gondii*-infected mice, suggesting that the decrease in Treg cell number caused by *T. gondii* infection would be associated with the impairment of Treg cell differentiation. Notably, TGF-β treatment upregulated pSmad3 expression, while TGF-β neutralization downregulated it. Therefore, TGF-β treatment could promote the differentiation of Treg cells through the TGF-β/Smad3 pathway, which may contribute to the amelioration of adverse pregnancy outcomes in mice infected by *T. gondii*.

However, limiting Treg cell differentiation was not the only explanation for the reduction in Treg cell numbers after *T. gondii* infection. It has been reported that reduced proliferation or enhanced cell death of Treg cells is also associated with the loss of Treg cells (Oldenhove et al., 2009). Our previous study showed that Treg cell apoptosis was increased in *T. gondii*-infected mice (Liu Y. et al., 2014). Here we also found that the proliferation of Treg cells was significantly decreased in infected mice compared with that in uninfected mice. Therefore, the decrease in Treg cell number in *T. gondii*-infected mice is due to decreased Treg cell differentiation and proliferation, as well as elevated Treg cell apoptosis. However, the proliferation of Treg cells did not change after TGF-β treatment or TGF-β neutralization, indicating that TGF-β administration failed to rescue the proliferation of Treg cells impaired by *T. gondii* infection. Therefore, the increase in Treg cell number induced by TGF-β treatment would be due to Treg cell differentiation, rather than Treg cell proliferation.

Cytotoxic T lymphocyte-associated antigen-4 and PD-1 are two important functional molecules on Treg cells (Freeman et al., 2000; Wing et al., 2008). Some studies have reported that TGF-β can induce the differentiation of human peripheral naïve T cells to Treg cells by upregulating CTLA-4 expression (Yamagiwa et al., 2001). Our previous study revealed that the absolute numbers of both CTLA-4^+^Treg cells and PD-1^+^Treg cells in the placenta and uterus decreased in *T. gondii*-infected mice (Liu Y. et al., 2014). In the present study, we found that TGF-β treatment increased the frequencies of both CTLA-4^+^ Treg cells and PD-1^+^ Treg cells, while treatment with TGF-β-neutralizing antibody reduced these frequencies. Therefore, TGF-β administration could strengthen the suppressive function of Treg cells by upregulating the expression of CTLA-4 and PD-1, which may contribute to preventing immunological attack of the fetus in *T. gondii*-infected mice.

Treg cells play a vital anti-inflammatory role during inflammatory responses by secreting IL-10 (Maloy et al., 2003; Connor and Anderton, 2015). TGF-β could induce Treg cells to synthesize IL-10 (Pyzik and Piccirillo, 2007). Conversely, the pro-inflammatory cytokine TNF-α is critical to the initiation and perpetuation of inflammation (Wing et al., 2008). This study showed that a placental IL-10/TNF-α imbalance, with the predominance of TNF-α, correlated with adverse pregnancy outcomes caused by *T. gondii* infection. TGF-β treatment corrected the IL-10/TNF-α imbalance in *T. gondii*-infected mice, and this corrective effect can be abrogated by TGF-β-neutralizing antibody.

## Conclusion

In summary, the present study demonstrated that TGF-β treatment could improve the abnormal pregnancy outcomes caused with *T. gondii* infection. The detailed mechanisms include upregulating the differentiation of Treg cells through the TGF-β/Smad3 pathway, strengthening the function of Treg cells, and modulating the cytokine imbalance in mice infected with *T. gondii*. Our work provides new insights into the improvement of pregnancy outcomes caused by *T. gondii* infection during early pregnancy.

## Author Contributions

XH conceived and designed the experiments. HZ, XL, and YJ performed the experiments. MZ, HZ, and LR analyzed the data. MZ wrote the paper and XH revised it. MZ, HZ, and XL made equal contributions to this paper. All authors read the final version of the manuscript and approved it for publication.

## Conflict of Interest Statement

The authors declare that the research was conducted in the absence of any commercial or financial relationships that could be construed as a potential conflict of interest.
